# Qualitative exploration of intrinsic and extrinsic factors that influence acceptability of semisoft vaginal suppositories

**DOI:** 10.1186/s12905-018-0657-2

**Published:** 2018-10-20

**Authors:** Toral Zaveri, Kimberly A Powell, Kate M Guthrie, Alyssa J Bakke, Gregory R Ziegler, John E Hayes

**Affiliations:** 1 Sensory Evaluation Center, College of Agricultural Sciences, The Pennsylvania State University, University Park, PA 16802 USA; 2 Department of Food Science, College of Agricultural Sciences, The Pennsylvania State University, University Park, PA 16802 USA; 3 College of Education, The Pennsylvania State University, University Park, PA 16802 USA; 4 College of Arts and Architecture, The Pennsylvania State University, University Park, PA 16802 USA; 5 Centers for Behavioral & Preventive Medicine, the Miriam Hospital, and Department of Psychiatry and Human Behavior, Warren Alpert Medical School of Brown University, Providence, RI 02906 USA

**Keywords:** Acceptability, Adherence, Sensory attributes, Focus groups, HIV, Sexually transmitted infections, Formulation, Vaginal microbicides, Formulation development

## Abstract

**Background:**

Vaginal microbicides are a promising means to prevent the transmission of HIV and other sexually transmitted infections, by empowering women to initiate use prophylactically when they perceive themselves to be at risk. However, in clinical trials, microbicides have shown mixed results, with the consistent finding that effectiveness varies substantially as a function of user adherence.

**Methods:**

Based on the assumption that adherence is driven, at least in part, by product properties that influence acceptability, we used softgel technology to develop vaginal drug delivery systems in the intermediate texture space between solids and liquids to overcome potential shortcomings of current dosage forms. Here, we used focus groups and surveys to determine women’s initial reactions (i.e., acceptance and willingness-to-try) for semisoft vaginal suppositories intended for HIV and STI prevention, with a specific focus on how perception of and preferences for vaginal suppositories may be influenced by product characteristics such as size, shape, and firmness.

**Results:**

Via focus groups, we identified intrinsic and extrinsic factors relevant to acceptability of semisoft suppository prototypes. Willingness-to-try depended on factors like intended functionality, anticipated leakage, type of sex, recommended frequency of use, type of sexual partner, and perceived risk. When handled ex vivo, shape, size, and firmness of suppositories communicated information about ease of imagined insertion and handling, perceived effectiveness, anticipated awareness and comfort of the product in the body. These impressions were partly based on prior experience with vaginal products.

**Conclusions:**

Sensory attributes appear to play a substantial role in women’s preferences and willingness to try the semisoft suppositories. Using these methods during preclinical development should help efficiently optimize a final product that is both biologically efficacious and preferred by women, toward a goal of enhancing adherence and effectiveness.

## Background

Vaginal microbicides are a promising HIV and STI prevention method that can be initiated and controlled by women. These microbicides contain one or more active pharmaceutical ingredients (APIs) and take the form of a gel, cream, foam, sponge, suppository, or film that is inserted vaginally [1]. Microbicides may be designed for pericoital insertion [2, 3] or regular, coitally unassociated use [4]. Heterosexual transmission of HIV to women has high public health relevance: in 2012, women represented 20% of those infected with HIV in the United States, and 85% of those infections arose from heterosexual contact [5]. In other parts of the world, women are disproportionately infected with HIV relative to men: in sub-Saharan Africa, women constitute 57% of all people living with HIV [6]. Due to gender inequality and other socioeconomic factors, women are not always empowered to negotiate safer sex practices, such as use of condoms. This problem is especially acute in unbalanced relationships where power dynamics are such that women do not have an equal voice in making decisions [7]. Vaginal microbicides would seem to be particularly advantageous in such situations, as women can initiate their use, with or without partner awareness. The potential for covert use (see [8, 9]) was explicitly explored in our study.

Microbicides with different APIs and in different physical forms, including the antiretroviral drug Tenofovir in the form of a intravaginal gel (1%), have been tested in multiple clinical trials [10]. The CAPRISA 004 trial conducted in South Africa found tenofovir gel reduced HIV acquisition by an estimated 39% overall, with a greater reduction (54%) among women with high adherence [2]. The VOICE trial conducted in Uganda, South Africa and Zimbabwe required daily application of the tenofovir gel, and women indicted difficulties associated with daily dosing, [4]; this study was ultimately discontinued due to futility [11]. Concurrent with the larger VOICE trial, an sub-study (VOICE-C) at one site in South Africa (of 15 total) used qualitative methods to explore reasons for low adherence; notably, biomarker evidence of nonadherence and self-report of nonuse were discordant [4]. Similar to the CAPRISA 004 trial, the FACTS 001 trial found some evidence that pericoital vaginal Tenofovir 1% gel was effective in women who reported higher use (72% of sex acts); unfortunately, these women only constituted 20% of the study population [3]. Therefore, pericoital use of 1% Tenofovir gel was not broadly effective at preventing HIV acquisition in the FACTS 001 trial, and this failure may be attributable to poor adherence [3]. More recently two double-blind placebo-controlled Phase 3 clinical trials of the daprivine vaginal ring showed significant reductions in HIV acquisition in African women. Again, this number increased when looking at groups with higher adherence [12, 13]. Collectively, these mixed results and in-depth efforts to understand reasons for failure of some of these trials highlight the critical importance of adherence if microbicides are to be successful as a means of prevention.

Reasons for poor adherence are myriad, including practical reasons (i.e., missed visits, lack of product replenishments, scheduling conflicts, forgetfulness, etc), social consequences (including stigma, and discrimination), partner complaints, knowledge of or beliefs about other participants non-use, side effects, fear of harm, and mistrust of stated research goals [4]. In the VOICE-C trial, women were also ambivalent about using powerful drugs when they had no illness, and a lack of demonstrated benefit was an important factor in non-use [4]. Other work suggests product features may also affect adherence. For gels, excessive leakage was a frequent complaint, leading women to not insert the full recommended dosage [14]. Some women questioned whether this leakage would reduce efficacy [15]. This is an key finding, as it shows that certain side effects related to physical form interact with women’s cognitions about efficacy, which may further reduce adherence. If women do not believe a product will work, it is unlikely they will use it. With solid rings, women describe themselves as adherent while also admitting they remove the ring for menses or intercourse. Non-adherent women suggested they were concerned men would oppose the ring or feel it during sex [16], and indeed some women have reported instances of violence when a male partner perceived a previously undisclosed ring [17]. Women also reported fears about inserting the ring and physical discomfort while using it, although with time, many women overcame these barriers [17]. Solid rings also have an additional drawback of being ill suited for anal sex. Based on National Survey of Family Growth (NSFG) data from 2011 to 2015, 1 in 3 American women aged 15 to 44 have engaged in heterosexual anal intercourse (HAI). Due to inherent differences between tissues in the vagina and rectum, as well as other environmental factors, it is unlikely one form could be optimized for use in both the vagina and rectum; still, some forms (like gels or suppositories) may be more easily used rectally by women who engage in HAI. Overcoming all barriers to product adherence (especially social and cultural barriers) may not be practical, but designing products that are both biologically efficacious and acceptable to women would seem to be a reasonable research goal [18], as acceptability is a major driver of adherence [19]. A product could be a very potent inhibitor of HIV infections but if a woman will not use it due to low acceptability (including physical characteristics), the product is functionally useless.

Prior work suggests acceptability is multifactorial and depends on packaging, side effects, safety, ease of use, the products’ impact on sexual pleasure, and sensory properties [20, 21]. These product attributes include appearance [22–25], smell [22, 23], taste, and textural properties that may affect sexual pleasure (how the product feels during intercourse) [15, 21], leakage (the propensity of the product to seep out of the body) [15, 23, 26], and vaginal coating [15]. Within the microbicide literature, there is a growing awareness that women conceptualize the efficacy of a product based on sensory properties of the product, and these beliefs, accurate or not, may influence use and effectiveness. For example, Morrow and colleagues found that differences in the surface appearance of intravaginal rings – matte versus shiny –were thought to indicate different degrees of porousness to users; critically, irrespective of how these rings actually release the API, a nonglossy (matte) finish was preferred [25].

Acceptability and meaning of sensory properties may vary based on intended use, as well as geographical region and culture, as sexual and vaginal hygiene practices are known to vary [27]. For example, during one microbicide gel clinical trial, young women in the continental United States and Puerto Rico gave opposite opinions on acceptability and likelihood of use based on their associations and perceptions of the additional lubrication resulting from gel application [28]: the additional lubrication was desirable for women in Puerto Rico as they associate the additional wetness with douching, whereas women in the US associated it with menstruation [28]. Likewise, Morrow and colleagues found that stickiness was undesirable in a lubricant, but desirable in long acting gels, as they would stay put and be “a better barrier” [25].

To address the diverse needs and preferences of women across different countries and cultures, multiple physical forms of intravaginal microbicides are in preclinical development and clinical trials [29, 30]. These forms include gels [2], rings [31, 32], tablets [33], and quick dissolving films [34, 35]. The different forms include both solids and liquids. Some breakdown inside the body and others need to be replaced periodically. Solid forms typically have the drawback of slow drug release, requiring a waiting period between microbicide insertion and coitus, which is often undesirable for the user [36]. Furthermore, women have been shown to react negatively to the “plastic” or glossy appearance of certain forms, including intravaginal rings and films [37, 38]. In contrast, liquid forms may be immediately efficacious, but often have a limited period of activity and must be reapplied prior to each act of intercourse (which may not be feasible for some users). In addition, users often complain of leakage with creams and “gels” (which are in fact liquids rheologically) [21, 28]. This leaves a broad design space of viscoelastic materials that have not been fully explored. Developing a product within this intermediate design space will give women another potential option if other forms do not meet her needs. Women’s preferences for the optimal vaginal state are diverse [39], so formulating diverse microbicide delivery systems may be an important strategy to help stem the spread of HIV and other STIs.

Accordingly, we have designed novel carrageenan based semisoft ovules that fall within the intermediate design space between solids and liquids and are capable of delivering pharmaceuticals into the vagina. Unlike other gelatin/fat-based vaginal suppositories, they do not melt upon insertion [40] but instead breakdown slowly, releasing the drug dispersed in the matrix. In the process of new product development, it is critical to understand how users interact with the product, how users conceptualize the product working, and perceive its effectiveness in the absence of additional information; these beliefs, accurate or not, are influenced by product attributes. Understanding this information prior to clinical trials may help prevent expensive failures of delivery systems that are functional but unacceptable. Better understanding of user preferences and perceptions early in the design process can guide formulation of products that endusers have confidence in and like, with downstream influences on adherence and compliance. Qualitative methods are well suited to exploring meaning generated by users, especially since they may identify factors that are more salient to users, versus those that are of primary interest for the research team [41]. Focus groups are a rich source for collecting this information, and can help guide product developers so they can focus on the attributes that are important to consumers [42]. To help our team identify the most salient user-identified product characteristics that drive acceptability and willingness to try, we conducted a series of focus groups where women examined prototypes of varying size, shape and firmness as well as explored various options for in-use features such as frequency of application, duration of protection, biological function, etc. We were primarily interested in understanding a) how these product attributes are linked to perceived efficacy, b) how preferences are formed, and c) what design parameters are critical for end user acceptability. Understanding how women make meaning regarding the function and efficacy of this novel dosage form can help modify the product design or product messaging to instill greater confidence, leading to better adoption of a dosage form.

## Methods

### Focus group design, protocol, and discussion

A total of nine focus groups were conducted; each group contained five to eight participants. A total of 57 women participated between February and April of 2012, and all completed a pre-focus group survey, one in depth focus group session, and a post-focus group survey. A female moderator (a highly experienced qualitative researcher) and female co-moderator (a biomedical engineer) facilitated the focus group discussions. A semi-structured moderator guide was developed ahead of time and tested on a practice group to collect feedback on the timing and sequence of questions prior to initiating the focus group study. The refined moderator guide was then used for all nine groups reported here. The study was conducted in a custom built qualitative research facility located in the Erickson Food Science Building on the main Penn State campus in University Park, PA. This facility includes tables, comfortable chairs, four overhead cameras and two overhead microphones linked to a digital recording system, and a one way observation mirror. All discussions were audio and video recorded digitally with redundant microphones and multiple overhead cameras. The audio recordings were transcribed verbatim using a commercial transcription service, and transcripts were checked against the audio and video recordings as needed by the research team. The transcripts were not returned to participants for comment or correction, given the sensitive and candid nature of the discussions.

All procedures were approved by the Pennsylvania State University Institutional Review Board (protocol #36943). Participants provided written informed consent and were reimbursed for their time.

### Study population

Eligibility criteria included being female, 18–45 years-old, heterosexually active (defined as having had vaginal sex with a man in the 12 months), and willing to have a frank discussion regarding preferences for vaginal products. All participants completed a survey of demographics, vaginal medication/product use, and sexual history; these are summarized in Tables 2 and 3. To recruit a more heterogeneous sample, various recruitment methods were used including campus email and fliers posted throughout the university and in local community venues such as fitness clubs and health clinics. Snowball recruitment was also used, in which we encouraged participants to tell women they knew who fit the participation criteria to contact the research team. Given the sensitive nature of the topic, women were scheduled in groups based on their age, as there may be differences in the nature of sexual relationships as well as vaginal product usage, and these issues may be more easily discussed among similar aged peers. Accordingly, women were grouped into one of three groups: 18–22, 23–30, and 31–45 years of age. Since we were recruiting primarily from a university community, the age ranges were selected to have a mix of undergraduate students (18–22), graduate students and postdoctoral scholars (~ 23–30), and staff and faculty (31–45). Sexual practices as well as vaginal product use also vary based on women’s home country [43]. To explore this, attempts were made to recruit women from different home countries and ethnicities. However, of women originally from outside the United States, only one group responded in sufficient number to make a focus group possible; thus, we conducted a separate focus group session for women from China. In total, 9 focus groups were run: 4 mixed race groups with younger women, 4 mixed race groups with older women, and 1 that only contained women from China. The majority of participants in the 8 mixed race groups self-identified as ‘White or Caucasian’ (see Table 2). Ideally, one may wish to conduct such research in populations at high risk for HIV and / or low negotiating power in sexual situations. However, as this was formative research centered on women’s experiences with initial prototypes, we used a convenience sample of sexually active women. Our goals were to understand how women link product attributes to efficacy, how preferences are formed, and design parameters that may affect user acceptability. While women of diverse backgrounds have different experiences, and may have divergent preferences, all women share certain some commonalities related to vaginal intercourse, vaginal hygiene, menstruation, and the use of related products.

### Data collection

#### Pre-discussion survey

After obtaining consent, but prior to initiation of the focus group, all participants completed a survey of demographics, sexual history, and vaginal product usage.

#### Focus group discussion

The focus group protocol and process was designed: a) to capitalize on participants’ previous experience with vaginal products and, b) to elicit their perceptions of the suppositories grounded in that prior experience. During the focus groups, we prompted for prior and current vaginal product usage as a means to establish comparisons between product history and the current prototypes developed for our study. Efforts were made to allow independent elicitation of vaginal products used by participants rather than our research team providing specific examples. We incorporated an *in-mano* (in the hand) manipulation protocol for suppositories of different size, shape, and firmness. This was followed by questions regarding perceptions, acceptance, and preferences for size, shape, firmness, and applicator/no applicator preference based on the sensory aspects of the suppositories. We followed up with a series of questions pertaining to usage in relation to: anticipated product residency (their desired frequency of usage and duration of protection); anticipated self-awareness of the product in the body (e.g., anticipated leakage of the material, lubrication, and covert use), including questions pertaining to adherence, residence, sexual activities other than vaginal intercourse (e.g., oral sex and anal sex), partner awareness, risk behavior, scenarios for product use, and likelihood of use in relation to these issues; and, willingness to try in combination with other STI medications, contraception, or lubrication, since suppositories can be used as drug delivery vehicles for varied and multiple indications. The concept of *covert use* was introduced by explaining that women are not always empowered to negotiate condom use due to socioeconomic or gender inequalities, cultural and religious beliefs, and/or cases of domestic sexual violence, and thus might need to use this product without a male partner noticing its presence. We then followed this explanation by asking how their perceptions, preferences, and/or willingness to try the product might change in relation to this information.

#### Presentation of microbicide prototypes

**Fig. 1:**
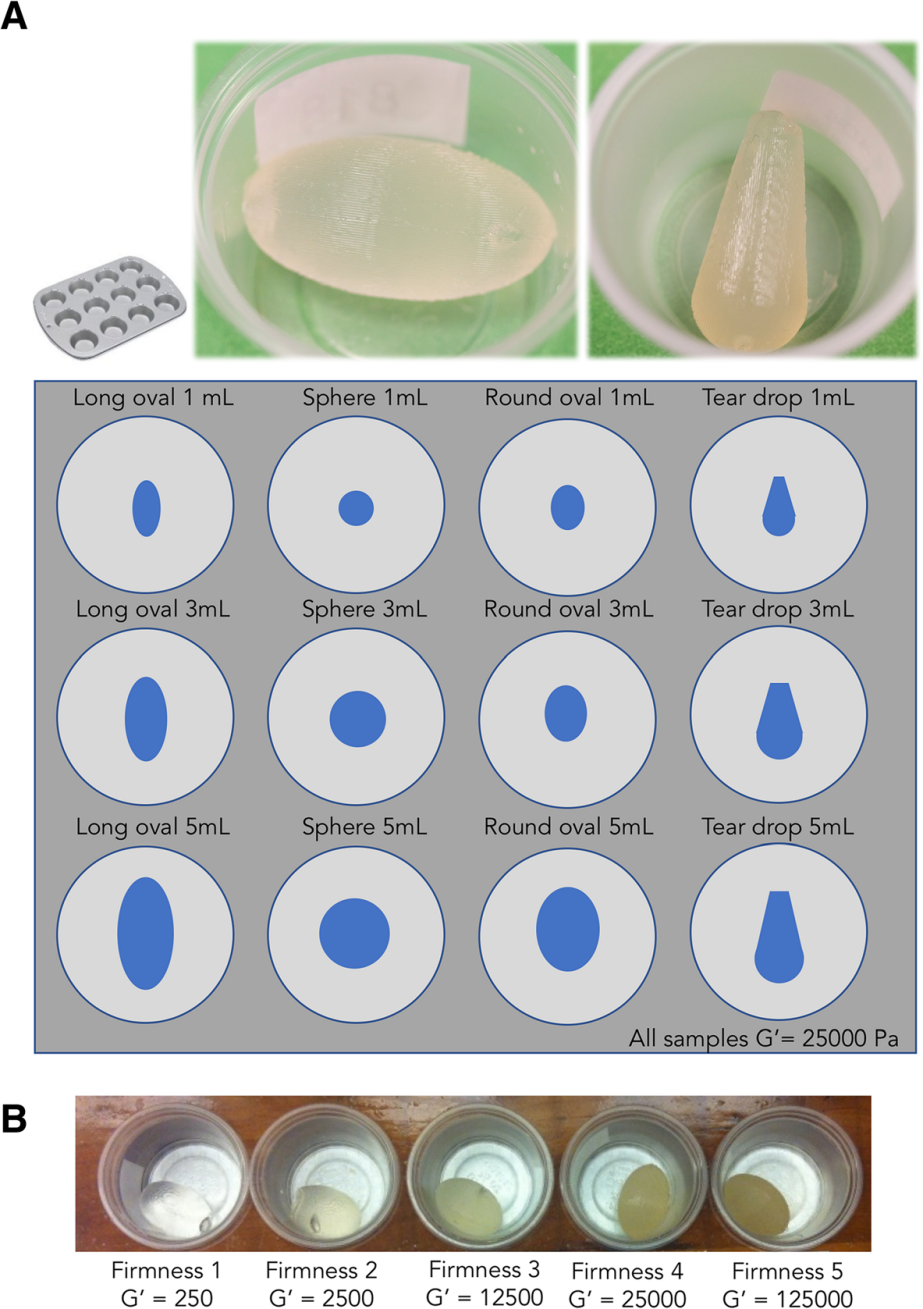
Illustration showing how suppository prototypes were presented to study participants. Each participant received her own set of samples to manipulate and examine. Panel **a** shows the ‘Shape and Size’ tray depicting the four shapes (Long Oval, Sphere, Round Oval, and Teardrop, from Left to Right) in three sizes (Size 1–3; 1 to 5 mL). Panel **b** shows the ‘Firmness’ tray depicting Round Oval in Size 2 (3 mL) prepared from gels with an increasing firmness level, from Left to Right. (G’ = 250 to G’ = 125,000 Pa at 25 °C). See text for additional details

After the moderator established rapport with the group, and the initial discussion of vaginal products was complete, each participant received a 12-cup mini-muffin tray (Fig. 1). The tray contained 12 semisoft suppositories of three sizes in four different shapes (details below). Individual suppositories were placed in translucent 0.75 oz. portion cups with opaque lids to allow sequential sample presentation. The four shapes were arranged in 4 columns with Long Oval on the left followed by Sphere and Round Oval with Teardrop on the far right. The three sizes were presented in different rows with 1 mL (referred to as Size 1 during focus groups) on the top, 3 mL (Size 2) in the middle, and 5 mL (Size 3) on bottom (Fig. 1). All the ovules in the shape and size tray were of the same composition and firmness (Formulation 3, Table 1). Details of the formulation were not provided to the participants, although they were told the prototypes did not contain any APIs. Women evaluated Size 1 first, followed by Size 2, and finally Size 3. The different shapes of a given size were evaluated simultaneously.

**Table 1:** Formulation for the different gels used to make suppositories for the focus group discussion

Firmness levels	Total carrageenan (% *w*/*v*)	Kappa (%)	Iota (%)	KCl (M)	Storage modulus (G’)(Pa)	Melting temperature (°C)
1	1	10	90	0.06	250	45
2	1	70	30	0.05	2500	53
3 ^a^	2	50	50	0.1	12,500	58
4	3	100	0	0.025	25,000	66
5	5	100	0	0.05	125,000	68

^a^ Formulation used to prepare samples for the size and shape tray

**Table 2:** Demographic Data for the Participants

	Number of women (%), *n* = 57
Age (years)	
18–22	17 (30%)
23–30	22 (38%)
31–45	18 (32%)
Race	
Caucasian or White	43 (75%)
Black or African American	3 (5%)
Asian	8 (14%)
South Asian (includes India, Pakistan, Bangladesh, Nepal, Bhutan, Sri Lanka)	1 (2%)
Other	2 (4%)
Employment Status	
Employed	24 (42%)
Student	17 (30%)
Unemployed	1 (2%)
Homemaker	5 (9%)
Student and Employed	10 (17%)
Highest Level of Education	
1 or more years of college, no degree	22 (39%)
Bachelor’s degree	17 (30%)
Master’s degree	11 (19%)
Professional degree	1 (2%)
Doctorate degree	6 (10%)
Marital Status	
Married	18 (31%)
Divorced	1 (2%)
Separated	1 (2%)
Never married	37 (65%)

**Table 3:** Vaginal Medication and Sexual History from the Pre-discussion Survey

Characteristic	Number of women (%) *n* = 57
Frequency of vaginal sex in the past 12 months	
Less than once per month	4 (7%)
2–4 times per month	31 (54%)
2–4 times per week	18 (32%)
More than 4 times per week	3 (5%)
Decline to answer	1 (2%)
Number of different male sexual partners in the past 12 months	
One	45 (79%)
2 to 5	8 (14%)
5 to 10	2 (4%)
Decline to answer	2(4%)
Types of sex women typically engage in	
Vaginal	17 (28%)
Vaginal & Oral	37 (65%)
Vaginal, Anal & Oral	3 (5%)
Types of sex women engaged in, in the past 12 months?	
Vaginal	16 (28%)
Vaginal & Oral	35 (61%)
Vaginal, Anal & Oral	6 (11%)
Frequency of lubricant use during vaginal sex	
Yes, all the time	5 (9%)
Yes, occasionally	18 (32%)
Yes, I have tried it	11 (19%)
No, I have never tried one	22 (39%)
Condom use during sex	
Yes, all the time	21 (37%)
Yes, only with someone new	2 (4%)
Yes, occasionally	8 (14%)
No, we use other methods of birth control	18 (32%)
No, we use other methods to prevent STI transmission	1 (2%)
No	5 (9%)
Yes, only with someone new & No, we use other methods of birth control	1 (2%)
Prior diagnosis of a sexually transmitted infection (lifetime)	
Yes	4 (7%)
No	53 (93%)
Frequency of STIs/HIV Screening	
Annually	19 (33%)
Once every 2–3 years	6 (11%)
Every time I change my sexual partner	19 (33%)
Never	20 (35%)
Decline to answer	1 (2%)
Number of vaginal deliveries (lifetime)	
One	9 (16%)
Two	4 (7%)
None	44 (77%)
Frequency of vaginal medication use for yeast infections or bacterial vaginosis	
Frequently	1 (2%)
Occasionally	13 (23%)
Once or twice	27 (47%)
Never used one	16 (28%)
Have you ever tried a douche?	
Yes	9 (16%)
No	48 (84%)
Frequency of tampon use?	
Yes, all the time	29 (51%)
Yes, occasionally	13 (23%)
Yes, I have tried it	8 (14%)
No, I have never tried one	9 (16%)
Use of spermicidal cream/gel for birth control?	
Yes	10 (18%)
No	47 (82%)

Following the evaluation and discussion of the first 12 samples in the shape and size tray, participants were then each presented a new tray with five increasing firmness levels; all five samples were the Round Oval shape with a volume of 3 mL (size 2). Samples exhibited a range from soft to firm (storage modulus (G’) from 250 to 125,000 Pa at 25 °C) (Table 1). Participants were told that this sample (Round Oval in Size 2) was not the researchers’ preference but served as a common point of comparison so that participants could focus on firmness levels. Participants were then asked to manipulate the product in her hand and make comparisons to the samples presented on the shape and size tray.

#### Post discussion survey

After all discussion was complete, participants filled out an exit survey outlining their preferences for color, smell, shape and applicator as well as rating the importance of various product attributes such as efficacy, convenience, and cost. The women completed the surveys individually while seated together in the discussion room. Since the questions within the survey were very personal in nature, the room was arranged to maintain a sufficient distance between participants to ensure privacy.

### Materials

Preparation and formulation of the suppositories, including their rheological characterization, is described elsewhere [40]. Briefly, to provide five levels of firmness (Table 1), gels were prepared by varying the total polymer concentration, the amount of potassium chloride (KCl), and the ratio of kappa and iota carrageenan in a mixture design. Commercial samples of kappa (κ) carrageenan (Gelcarin NF 911, Batch Number 10707011) and iota (ι) carrageenan (Gelcarin NF 379, batch number: 10514011) were kindly provided by FMC Biopolymers (Philadelphia, PA, USA).

### Data analysis

Analysis of the focus group transcripts were informed by a qualitative approach to thematic analysis, which allows for both inductive categorization of themes that build towards a theory of explanation regarding the phenomena and population under study, as well as for categorization of themes based on existing research [44]. A list of themes was generated based on a priori issues that were part of the moderator guide, the existing literature on microbicide acceptability studies, as well as issues that women brought up during the group discussions. The transcripts were coded based on this list of themes. To ensure coding agreement and to develop and ensure inter-coder concordance, two independent researchers coded and discussed the first five transcripts (of nine total). Based on the agreed coding rules, the remaining 4 were coded by one researcher. The coding tree had 14 top level nodes, with one or more secondary nodes nested within each top level node; some but not all secondary nodes had tertiary nodes nested within them. For example, all statements regarding Firmness level 5 were coded as 1.3.5 (1, Product Characteristic > 3, Firmness, > 5, level 5) while statements about adherence were coded as 7.2.3 (7, Willingness to use > 2, dosing regimen > 3, adherence) and statements about partner awareness were coded as 11.1 (11, covert use > 1, partner awareness). The mutually agreed upon codes were then entered into NVivo qualitative data analysis software (QSR International Pty Ltd. Version 9, 2010) and coded transcripts were queried for the themes individually or in combination.

## Results

Topics from the transcript analysis are detailed here. These are separated into three major themes: a) Willingness to try, b) Covert Use, and c) Perception of and preference or size, shape and firmness.

### Willingness to try

Women’s willingness to try depended on six inter-related factors: 1) product function; 2) anticipated leakage from the suppository; 3) type of sex; 4) frequency of use as a perception of product effectiveness; 5) type of sexual partner; and 6) risk perception. Reasons and explanations for each are described below. Due to the age dependence of some of the factors listed above, such as number and type of sexual partners, willingness to try varied with the age of the participants. The need for the product in ‘special situations’, such as traveling to parts of the world where HIV is believed to be endemic, was also raised by participants.

#### Product function

Willingness to try largely depended on their proposed biological function, prompted by the focus group protocol: specifically, the ability of the suppositories to act as a contraceptive, for HIV prevention, for prevention of other STIs (e.g., chlamydia, gonorrhea), as a vaginal moisturizer, or some combination of these. In each group, a majority of women were willing to use the product for prevention of STIs such as chlamydia, gonorrhea, herpes, and HIV. Some did not feel the need in their current circumstances but were definitely willing to try the product should the need arise. This is illustrated by statements from the following participant:

I was thinking about it like way down the line. If it prevented chlamydia or gonorrhea that would be nice, and in other parts like in other times in my life that might’ve been something I would use it for. If you can find one that will prevent transmission of HPVs, especially and HSV—so herpes and genital warts I mean because those aren’t curable. Those are much more like in the category of HIV, and the other thing too is that condoms are kind of imperfect at blocking the transmission of those so if it gave me an extra layer of protection against things that condoms can’t completely protect against, I would absolutely use it. (23 years old, Caucasian)

Although a multipurpose product (i.e., one that would combine protection against a multitude of STIs) was strongly favored by a majority of the women in all the groups, reactions to a combined STI prevention / contraceptive product was mixed. Sentiments were expressed as: “I think if you can combine birth control and STD/HIV protection this product would be incredibly popular.” However, women currently using the NuvaRing®, birth control pills, and condoms were unwilling to switch to a contraceptive suppository for reasons including the need for frequent application and increased discharge compared to NuvaRing®, reduced ease of use compared to pills, and increased exposure to hormones compared to using condoms. A few women indicated they would prefer the suppository to contain spermicide (as opposed to hormonal birth control) or said they would use a suppository containing spermicide as a backup method of contraception along with condoms and pills. A few women were willing to use it as a contraceptive for reasons such as preference over condoms or dissatisfaction with current birth control products on the market.

Of women aged 18 to 22, a majority endorsed a suppository that combined prevention of HIV and other STIs with a lubricant. Among all ages, a majority of the women expressed a willingness to use the suppository as a vaginal moisturizer or lubricant, with some suggesting that suppositories would be more convenient to carry, as compared to a bottle or tube of lubricant. One exception was in the focus group with Chinese women. A majority of participants indicated they preferred natural lubrication and didn’t feel the need for such a product; this finding aligns with other studies suggesting the preferred amount of lubrication during coitus may be culturally dependent [43].

#### Frequency of use as a perception of anticipated product effectiveness

The women had different preferences for how often they would like to use the product, depending on their lifestyle and beliefs about product effectiveness over time. Stated preferred frequencies (in order of most to least popular) were: weekly, as needed just prior to sex, every 2–3 days, with the lowest preference for daily use. Daily insertion, as well as a 2–3 day schedule, were least preferred, as women felt it would be cumbersome to remember every day, especially for women who already used birth control pills and “did not want one more thing to worry about.” Weekly use was mentioned as being “easy to keep track of.”

Even with my lifestyle, looking at the birth control aspect of it, if it was between every time I’m going to be having sex versus every three days I would still choose the every three days. I don’t like the idea of having to stop. That’s why we don’t use condoms, cuz I didn’t like to have to stop to do that every single time, especially if you’re having a really good night, and you do have it a couple times. Having to stop and do that each time is just—it’s frustrating and it’s irritating. I don’t like it, so even then I would rather have to put it on my calendar. Okay, every Wednesday and Saturday this is what I—the days that I need to take it and set my cell phone. I would rather go that route than every single time. (33 years old, Caucasian)

Preferences for frequency of application were also based on the API the suppository would carry; for example, if the product contained a yeast infection medication, women preferred to use it only as needed or prescribed:

I mean, for me it really depends on what is in the vehicle, I guess, like whether it’s for HIV protection I would like a daily sort of thing. If it’s for sort of like a probiotic or—I’ve never dealt with yeast infections or anything like that, so I’m a little foreign to that, but I feel like those things I could be a little more hands off, and be like whatever, just put it in there, and it’ll work sort of thing, like it’s not an active like—there’s not like an active assault sort of on my body’s health, and it’s more of a preventative treatment then it isn’t—I guess I wouldn’t need to have contact with the product as much, if that makes sense. (26 years old, Caucasian)

Generally, if the suppository carried medication to prevent transmission of HIV or other STIs, women preferred weekly or 2–3 day schedules based on the frequency of their sexual contact. There was greater confidence in the product if it were applied more frequently:

For hygiene reasons I would not want to have to take it out. I would much prefer it to disintegrate, and I would personally prefer an either daily or max (maximum) weekly insertion because if I was doing it once a month I wouldn’t feel as confident that it was working. (19 years old, Caucasian)

#### Perceived leakage

Concepts such as perceived wetness and potential leakage played an important role in willingness to try. In half of the groups, some women said they didn’t mind leakage or discharge if it were for a limited time after insertion or an indication of viral protection, although some preferred that discharge from the product would be similar to vaginal secretions as this would aid in low partner awareness and covert use of the product. However, in most of the groups some women said they would not use the product if there was product discharge, especially if the discharge would be chunky. A few women pointed out that discharge of the product could also raise their suspicions regarding the effectiveness of the product, as the user could not be sure if she was excreting the vehicle or the drug itself. Discharge in amounts that would require the use of panty liner was also ‘a deal breaker’ for some women, consistent with other quantitative data [9].

#### Type of sex

Willingness to try also depended on the imagined type of sex (e.g. vaginal, oral, anal). Oral sex in particular elicited mixed reactions. Women expressed concerns about exposing their partner to the API if receiving oral sex, and the product possibly having a taste or smell was a major issue: one participant referred to this as “the ick factor”. A few women felt that if the medication was not toxic, and had no taste, then their partner wouldn’t mind it or they wouldn’t mind using the product while receiving oral sex.

#### Risk perception and sexual partner type

Willingness to try was heavily influenced by perceived risk. Risk perception was intimately tied to sexual partner type. Based on the survey prior to the focus group, a large majority of participants (79%) reported having just one sexual partner in the past 12 months, and a few of those explicitly stated during the discussion that they did not believe that they were at any risk for HIV, as they were with stable partners. Willingness to try when engaging in risky sexual behavior, such as with a new sexual partner, depended on women’s confidence in the product. A few women in stable relationships who did not currently perceive themselves currently at risk stated they would use this product with a new sexual partner, while others said they would not trust this product with a new partner, and would therefore prefer to use a physical barrier such as condom, using the suppository for additional protection. Some women in long-term relationships also expressed interest in the product as a backup method along with condoms. These beliefs were closely related to risk perception. Type of sexual partner and risk perception also influenced women’s usage frequency preferences. In more than one focus group, participants felt women in long term relationships or with a consistent partner could follow a different schedule compared to women who engage in casual sex or are under the threat of being sexually abused or raped. Women aged 18 to 22 discussed scenarios such as sexual assault and suggested that this would affect desired frequency and residency of the medication.

We often talk a lot about like put it in and go to a party, like have a good time, and whatever happens happens, but like there are just unexpected things that could happen. People get raped and assaulted and things like that, so it would be good to have it in, and you’re protected. (21 years old, African American)

Notably, some participants felt that HIV may have been a threat in the 1980s but wasn’t really a threat today. Conversely, women perceived a greater risk for contracting other STIs, especially women aged 18 to 22, who did not completely trust condoms or their sexual partners. Some indicated a high willingness to try if their life situation changed, partner changed, or if they were traveling to areas where they believed HIV to be a bigger threat. Women aged 22 to 45 frequently engaged in projection, mentioning they wouldn’t need such a product now but felt it would’ve be beneficial when they were younger as well as being beneficial for younger women today, as there was a common belief that teenagers and young adults engage in risky sexual behavior, such as casual weekend sex, and are, therefore, at greater danger of HIV infection.

### Covert use

Relative to their own situations, women were split about covert use. In all the groups, some women felt that it was not necessary to let their partner know they were using such a product to avoid having an uncomfortable discussion, or to raise potential “trust issues.” In half the groups, women said they wouldn’t mind sharing the information with stable partners or with partners they could trust. The remaining women stated that they had no need to use this product in their life, reasoning that their stable long-term relationships did not put them at risk of contracting HIV. It should be noted, however, that three of our participants work in health-related fields (e.g., nursing, social work), and shared data, stories, or statistics with other participants pertaining to local needs for covert use.

Generally, opinions about women’s preferences in situations much different from their own – such as geographic regions at-risk of HIV infection, socio-economic status that would put one at risk of being unable to afford the product, or women who were sexually active with multiple partners — were based upon their own experiences and perceptions of other women’s lives and needs, whether correct or not. Here is such a statement from one participant:

Even if you’re not in a relationship where it’s not like a one-night stand, say if you got raped and you know, the nice thing with this, even if you’re not sexually active yet, and you’re taking that—you started this like at age 13. Say something happens, and you get date-raped or something, this is one less thing you have to worry about. (20 years old, Caucasian)

Participants identified multiple issues they felt would need to be considered when designing a product for covert use. To avoid raising suspicion, they suggested the microbicide should be used without an applicator and minimal packaging, which would also necessitate a firmer product for manual insertion. Another suggestion was that the product should be small in order to have less leakage, be quick-dissolving, and avoid being felt by the partner. Some women said softer firmness levels (1–3) would feel like the vaginal wall or secretions and hence help with covert use. Considerable leakage or particulate discharge was perceived as interfering with covert use.

I think that the smaller Size 1 or 2, partners that I’ve had would not really notice it. (…) You know, 1 and 2 felt very smooth so the partner may just think it’s your own body’s fluids. (36 years old, Caucasian)

Women also felt storage of the product may be a great concern for other women, in terms of both access and visibility: a product that required refrigeration risks being seen and therefore cannot be used covertly; and a product requiring storage at clinics may be difficult to access for many women, leading participants to suggest a product that stores at room-temperature and has a long shelf life.

### Perceptions and preferences for shape, size and firmness of suppositories

As expected, product shape, size, and firmness influenced women’s acceptance of vaginal microbicides. During the focus groups, the Long Oval shape received the most positive reactions followed by the Round Oval and Teardrop. Most women disliked the Sphere shape. Women preferred shapes that had prior associations with vaginal products. Unfamiliarity and associations with products not normally associated with vaginal insertion were generally viewed negatively. The Long Oval’s resemblance to numerous other vaginal products on the market was explicitly stated as a reason for preferring it. Such comparisons include yeast infection suppositories and tampons. A small number of women did not like the Teardrop because they were either unfamiliar with this shape or associated it with objects such as a vibrator or other objects that they do not associate with vaginal insertion, giving examples like a “fishing lure” or “chip of ice.” Many women said that they associated the Sphere with “bouncy balls,” “gumballs,” “beads” and “marbles”. They indicated these associations were a barrier in their willingness to try the product.

During the focus group discussions, Size 2 (3 mL) was the most preferred size and firmness preferences varied, depending on whether or not participants would use an applicator for product insertion. Four primary themes emerged from discussions pertaining to suppository preferences that help illuminate the reasons behind shape, size, and firmness preferences. These were: 1) handling and imagined insertion; 2) perceived effectiveness related to physical properties of the suppository; 3) awareness and comfort of the product in the body; and 4) prior vaginal product use.

#### Ease of handling and imagined insertion

Women handled and manipulated all prototypes in the hand, but they were not inserted vaginally, as this was a preclinical study. In all groups, the reasons women gave for preferring the Long Oval included ease of handling and imagined ease of insertion, described by a few as the resistance to breaking during insertion. (Similar to the Long Oval, Round Oval was preferred due to imagined ease of insertion. The Teardrop shape elicited the most mixed reactions, with conflicting views on imagined ease of insertion: women in over half of the groups thought the teardrop shape would be easy to insert with its rounded bottom and pointed tip; however, in the same groups there were other women who also perceived this shape as difficult to insert. Reasons given for included confusion about the direction of insertion, squirting out of the hand, or a belief that it was not as sturdy as the other shapes due to the asymmetry. In most groups, the sphere was the least favored shape due to perceived difficulty of handling and inserting a rounded object. Many women discussed the resemblance of the sphere, especially in the larger size to a “gumball” or “bouncy ball,” candidly expressing concerns marked with humor about the ball “jumping around” or falling out and bouncing on the floor.

As mentioned above, Size 2 (3 mL) was the most commonly preferred size; there were explicit statements that it would be easier to insert than Size 1 (1 mL) as they thought Size 1 would require an applicator for insertion. A few women preferred Size 3 (5 mL), as they felt it had enough surface area to hold onto while imagining trying to insert it with fingers.

In terms of firmness preference, half of the participants made a connection between firmness and time required to breakdown in the body and would prefer a less firm product that broke down more quickly. Firmness preferences were strongly influenced when the concept of an applicator for product insertion was introduced. In all groups, firmness level 1 was said to be very soft, difficult to handle and fragile; yet, if given the option of an applicator, a few women stated that they would prefer Firmness 1 as they believed that it would break down quickly, absorb into the body, and provide instant protection.

Number 1 (Firmness 1) actually doesn't freak me out as much. I mean it did break pretty easily, but also I would kind of need to know more about how like the drug is absorbed because this, Number 1, seems like it would be absorbed maybe quicker or easier than—I mean obviously number five (Firmness 5). I don’t know. I don’t know how—I feel like the firmer it gets, the longer it would take to get into your system. I mean I don't know anything about the way drugs get into your system from your vagina. (22 year old, Caucasian)

In the absence of an applicator, a preference for a firmer product was expressed in more than half of the nine groups, with stated preferences varying between Firmness 2 to 4 depending on their perceived ease of handling and inserting each of the prototypes. These reactions and responses suggest the most acceptable version of this prototype would be very different depending on whether or not it is delivered with an applicator, in agreement with other quantitative data [45].

In the prototypes evaluated here, color and translucence were confounded with firmness (see Fig. 1a). That is, as the amount of carrageenan increased in the firmer ovules, translucence decreased and the suppositories appeared slightly yellow in color. A few women associated the clear appearance of Firmness levels 1 & 2 as pure and free of chemicals and preferred these due to the idea that the vaginal discharge will be similar to natural mucus secretions.

#### Perceived product effectiveness related to physical properties of the ovules

The Long Oval also looked the most medicinal to women, with comparisons made to vitamin tablets, fish oil capsules, or medication more generally. Preferences for the Long Oval and Teardrop shapes over the other two shapes were related to their larger surface area, as women believed surface area would be related to the rate of medication dispersal.

If you have sort of like a medication or whatever that’s being administered through these things, it seems like if you had an increased surface area you’d have a quicker dispersal of that medication or probiotic or whatever else you’re trying to administer. It seems to me like that would be more fast-acting. Whether that’s actually true or not, I don’t know, but that would be my perception. (26 years old, Caucasian)

A few women believed there was a relationship between the physical size of the ovules and the amount of medication contained within an ovule, associating this with product efficacy. Despite of being explicitly instructed that each size could carry the same amount of medication, a few women did not believe, or said other women would not believe, that Size 1 would carry enough medication to be effective against HIV. In over half the groups, some women believed Size 2 or 3 would potentially carry more medication and be more effective. Women also expressed size based on *perceived* duration of protection. For example, for daily and weekly applications, they would prefer Size 1, whereas for weekly and monthly applications, they would have more confidence in Size 2 based on the amount of medication they believed it could hold. Here is a description from one participant that typifies this common belief:

I feel like with the smaller ones, I would almost be worried I wasn’t getting enough of whatever it’s supposed to be, medication or whatever it’s supposed to be. That also depends on how often you’re using it, but I would almost feel like the smaller ones, you’d insert it, and then kind of be like it’s so tiny, I would almost be concerned I wasn’t getting what I was supposed to be getting out of it. Even if it could still have the same amount, would just be more concentrated, I think it would be a mental thing. (25 years old, Caucasian)

Some women expressed a fear that certain shapes, sizes, or firmness levels might fall out of the body. For example, because of the shape of the sphere, some women expressed a fear of the product rolling and falling out of the vagina. Also, some felt that Size 1, regardless of shape, had a higher likelihood of falling out of the body during daily activities or sexual intercourse. Regarding firmness (see Table 1), a few felt that Firmness 5 might fall out of the body. This belief may have been linked perception of the amount of time required to breakdown within the body:

(Firmness) 5 for me (is) kind of heavy feeling, which is not—I feel like that would fall out. Feels like not made of gel, like made of—I don’t know. It just feels solid, whereas (Firmness) 4 feels like a gel, and the rest feel like gel. I just think (Firmness) 5 is too thick. (19 years old, Caucasian)

#### Perceived comfort and bodily awareness

Based on evaluation in the hand, imagined comfort was generally associated with anticipated awareness of the product once it was in the vagina. Women’s beliefs about of how similar or different a shape was to the natural shape of the vagina influenced preferences. The Long Oval, Round Oval, and Teardrop (in some sizes) were perceived as being comfortable once inserted. The Long oval, in particular, was viewed as a similar and appropriate shape for the vagina, described as “comfortable” or “natural”. In contrast, some women perceived the round sphere as uncomfortable.

… I just agreed about the circle feeling weird because even though it’s small enough that it probably wouldn’t make a physical difference, when I imagine my vagina it’s the long tunnel and I feel like a sphere being in the middle of it would corrupt the edges of it, so I would definitely prefer either a teardrop — actually I’m leaning more towards the long oval and maybe because it does resemble the little circle that I’ve seen on a Vagisil box. The teardrop looks too much like a non-standardized shape, and I don’t know why that matters with administering medicine, but still that same sort of long, thin shape I think would work best with my conception of inserting something into my vagina. (22 years old, Caucasian)

Women discussed about tradeoffs between comfort inside the body and perceived effectiveness when discussing size. Size 2, the most commonly preferred size, was perceived as a happy medium between being comfortable inside the body and carrying sufficient medication. Although women believed that Size 3 would be more effective as they thought it could potentially carry more medication, a small number of women thought that its presence would be felt inside the body to the point of being painful. Size and firmness appeared to interact and shape women’s opinions, as this perception was more noticeable when women discussed firmness preference. Size 3 was perceived to be much more uncomfortable with increasing firmness. In general, at least one woman from each of the groups disliked Firmness at levels 4 and 5, as they believed that it would be felt in the vagina and be uncomfortable and/or noticeable to their partners during intercourse.

As previously discussed, potential for leakage and discharge was a key concern. After discussing potential discharge and leakage from suppositories, several women changed their previously stated preferences toward softer firmness levels (Firmness 1 or 2) in order to avoid chunky discharge or toward a smaller size (Size 1 or 2), believing that Size 3 or larger might lead to more leakage.

### Post discussion surveys

**Fig. 2:**
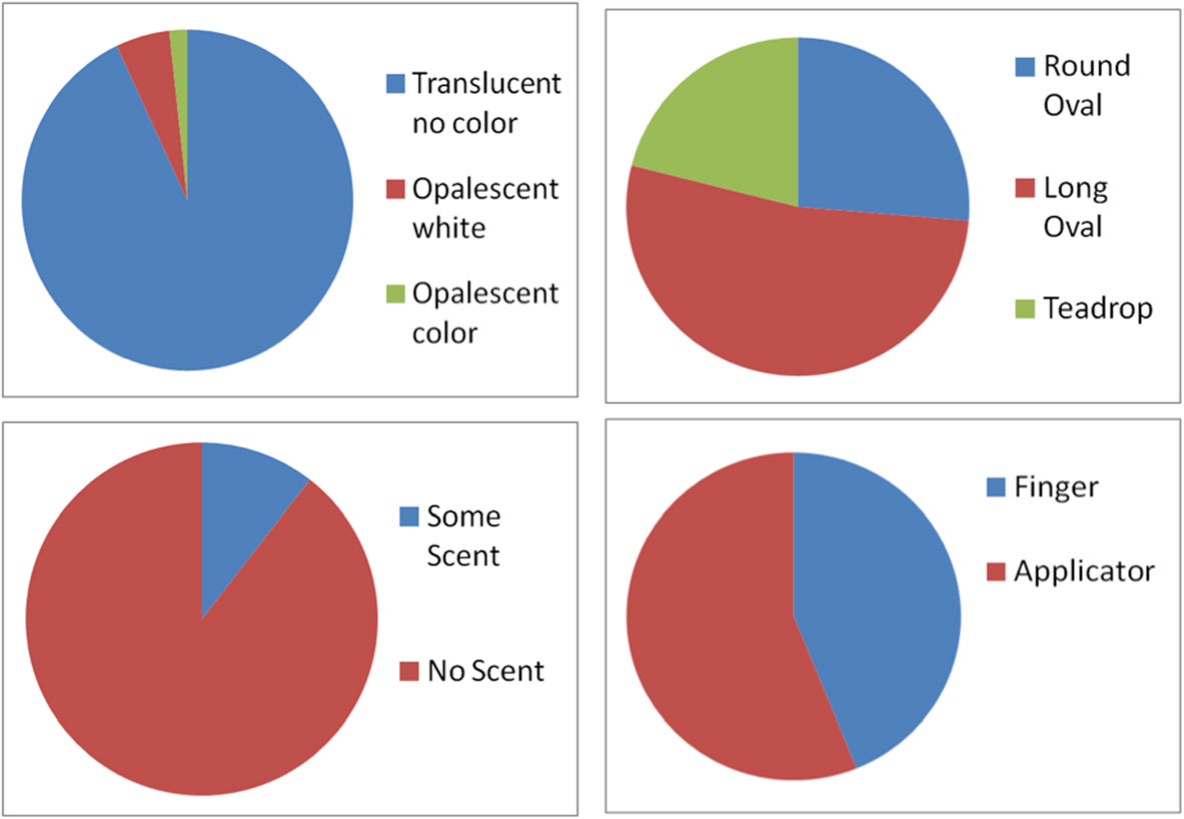
Summary of preferences for color, shape, smell and applicators, based on individual surveys collected after the focus group discussions

Surveys collected after the focus group discussions generally mirrored the findings from the focus group discussion: ~ 53% of the women (30/57) preferred the Long Oval shape, 26% (15/57) preferred the Round Oval, and 21% preferred the Tear Drop (12/57) with a few women indicating preferences for more than one shape (Fig. 2). When given a choice in the post FG survey, 56% of women (32/57) (Fig. 2) said they would prefer an applicator. Notably, 93% women (53/57) indicated they would prefer a product with no color, translucent like vaginal fluid, and 90% women preferred a product with no scent.

## Discussion

It is important to understand how women draw meaning regarding products based on their appearance, and how they conceptualize the product working, based on other products they may have used [37]. Comments made by participants during the focus groups described here may not be scientifically accurate. For example, even when specifically told that all sizes could carry the same amount of medication, some women indicated they would have more confidence in the efficacy of larger sizes. Likewise, women had beliefs regarding the shape of their vagina, so women conceptualized that the Long Oval shape would be the best fit, irrespective of the reality that all the shapes presented here would fit equally well given the normal elasticity of the vagina. Curiously, women who preferred more natural products believed that the more translucent ovules were chemical free, and they would be more willing to use them, even within the explicit context of a discussion of a pharmaceutical delivery system. Thus, we caution product developers and formulation scientists to be aware that factually inaccurate beliefs may still drive acceptance or rejection of new products.

**Fig. 3:**
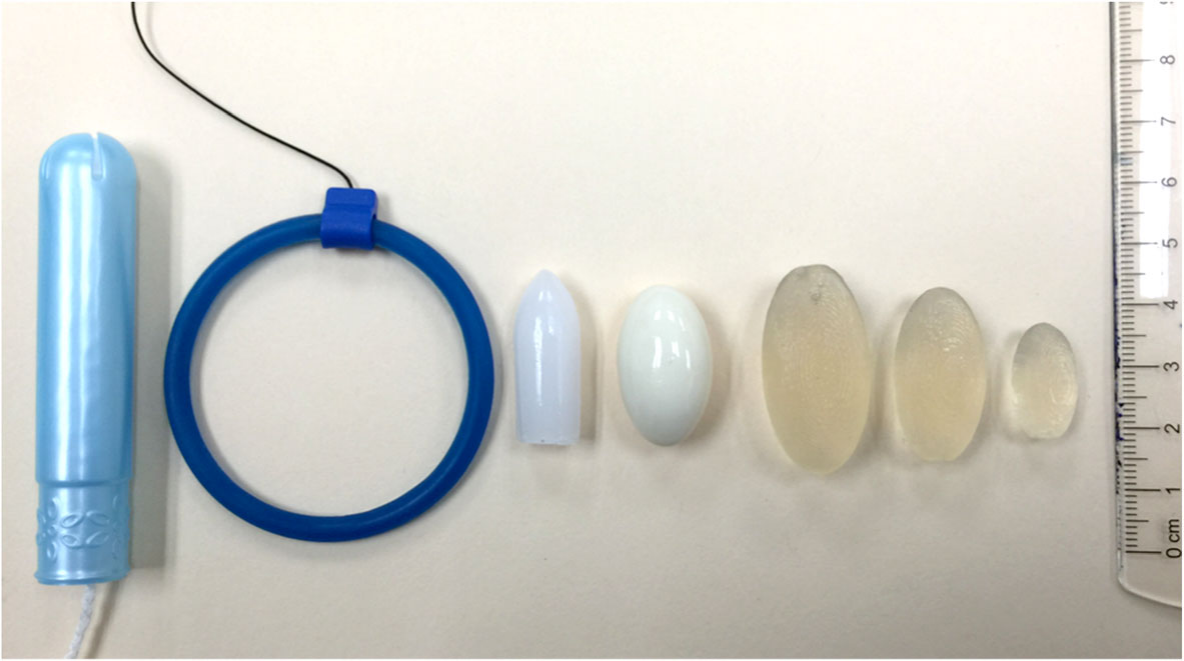
Comparison of prototype softgel suppositories to commercial vaginal products described by participants in the focus groups. From left to right: store brand Regular size tampon with plastic applicator, NuvaRing® intravaginal contraceptive ring (the clinical device is transparent white; a blue demonstration device without any API identical in size and shape is shown here), Noroform®- Feminine Deodorant Suppositories, Monistat® 3 Vaginal Antifungal Ovule Inserts, Size 3 (5 mL) softgel, Size 2 (3 mL) softgel, and Size 1 (1 mL) softgel suppository

Elsewhere, prior vaginal product use has been shown to influence microbicide acceptability [46–48]; however, few reports detail direct comparisons between other vaginal products and microbicide prototypes or explore how the prior product use directly influences preferences of various microbicide prototypes (Fig. 3). We show how prior vaginal product use influenced the perception of appropriateness and thus preferences of size, firmness and shape of suppositories. While designing the product for different world markets, it would be beneficial to review the vaginal products used within the local market, as vaginal products vary greatly across regions and cultures. With the carrageenan semisoft suppositories described here, minor formulation changes and mold modifications could be used to alter the physical properties of the suppositories to meet the needs of the local market.

Willingness to try this novel dosage form was driven by factors such as function, leakage, type of sex, frequency of application, duration of protection, risk perception and type of sexual partners of study participants. While some of these factors have been widely studied in the context of microbicide acceptability such as leakage [18, 20, 21, 36, 49] and risk perception [50], biological function [51, 52] other factors such as timing of application [53, 54], duration of protection [51, 54], type of sex [50, 55], partner type [56] and specifically their association with product’s physical attributes are still not well studied. Focus group discussions provided us with insights into how attributes such as size and firmness must be optimized to minimize leakage, instill confidence regarding efficacy for the specified time period, be suitable for different types of sex, and possibly allow for covert use. Even if certain parameters cannot be modified as per user needs, these findings may inform messaging and education efforts to increase adherence in subsequent clinical trials [57]. Here, women expressed a need for a product to be tasteless so that it is not noticeable to their partners, especially during oral sex. Although carrageenan itself is inherently tasteless, we should note APIs contained within the suppository may still be noticeable when tasted (e.g., bitterness and chemesthesis will likely contribute to the flavor profile of the product [58, 59]).

Conducting acceptability studies during preclinical development has the potential to help guide final product design, toward a goal of designing a product that is both biologically efficacious as well as preferred by women, thereby enhancing adherence and improving real world effectiveness (e.g., [54]). This work was the first of several acceptability studies conducted as part of ongoing research in this area. Based on insights from the qualitative data described here, we subsequently conducted multiple, large ex vivo quantitative tests to narrow the product design space in terms of physical attributes [24, 45, 60]. The focus group results greatly informed the design of subsequent quantitative tests to help further optimize the product design space. These optimization efforts have been a good example of the use of mixed methods in preclinical microbicide design. To quantitatively determine optimal size and firmness in a subsequent study, we asked women to evaluate suppositories of varying size and firmness *in mano* (in hand) in isolated sensory booths and rate imagined ease of insertion and willingness to try. Insights from the focus groups described here were instrumental in determining the appropriate range of the attributes to present in such quantitative tests. Since Long Oval was the most favored shape in the focus groups, it was selected for efforts to optimize firmness and size. Based on the divided opinion of women on use of applicators in the focus group discussions as well as differing firmness and size preferences based on presence or absence of applicator, we determined the optimal size and firmness in two separate groups of women: one group was asked to imagine insertion with an applicator and the other group without [45]. Consistent with the focus group data, women gave higher willingness to try scores to larger and firmer products without applicators, as compared to with applicators.

As product preferences were influenced by prior vaginal product use, we then designed a second generation of shapes (tampon and bullet) based on existing vaginal products such as tampons and Vitamin E suppositories. These new shapes were compared with the more preferred shapes from the focus groups (Long Oval, Round Oval, and Tear Drop) in a large scale quantitative study [24]. Women rated the five different shapes for preference, size, and firmness, and their ratings were correlated with length and thickness of the different shapes. While determining the correlation between size and firmness perception and product dimensions for teardrop shape, data from focus group discussion indicated that women would prefer to hold the thicker end of the Teardrop, and product dimensions were modeled accordingly [24]. Based on the consistent negative response to the sphere in focus groups, the sphere was eliminated from optimization with second-generation shapes such as bullet and tampon [24].

To quantify how different use parameters such as leakage, wait time after insertion, partner awareness, and duration of protection affect women’s willingness to use the product, Primrose and colleagues conducted web-based conjoint analysis with 302 sexually active women [9] . Consistent with the findings described here, there was strong interest in the multi-functionality of the suppositories for prevention of STIs and HIV as well as for contraception; that is, multipurpose prevention technologies; MPTs. Women both in focus groups and the quantitative conjoint analysis study expressed strong preference for clear/translucent products (Fig. 1), which do not produce discharge and thus are not noticeable by partners. There was also interest in fast-acting products, which also continue to work for 2–3 days after insertion [9].

## Limitations

Focus group methods may not always be well suited to collecting data of a sensitive nature, such as vaginal product usage and sexual behavior, as was done here. Accordingly, it seems likely there was a selection bias for participants who opted to participate in our focus groups. Higher than expected willingness to try the product may be an artifact of opting to participate in the study rather than a need to use such a product. About 75% of the women in the focus group discussion were Caucasian and 60% of women had a Bachelor’s degree or higher. Hence, we must caution that the opinions and beliefs raised here were expressed by primarily Caucasian women on or near a college campus, and the opinions expressed may vary based on location and ethnicity of participants. Future work is needed to extend these finding to other populations in the United States, and as well as regions like sub-Saharan Africa, where many of the current and past clinical trials have been conducted. Finally, we cannot understate the importance of perceived risk in microbicide acceptability; the majority of women here were in stable single partner relationships with low perceived risk, so more work is needed to extend these findings to higher risk groups.

Here, we have attempted to research and understand user preferences and perceptions early in the design process to guide formulation of a product that users have confidence in and like. Having said that, women in our focus groups only handled the products ex vivo; we fully acknowledge that preferences and perceptions may differ when women insert similar products vaginally and/or use them during coitus. Collection of additional acceptability data in vivo as part of clinical trials is needed.

## Conclusions

Conducting preclinical product design research with potential end-users allows critical characteristics of formulation parameters to be understood early in the development process. This study points to the need to understand both observable product parameters (size, shape, firmness) that women will be willing to try for various sexual health indications, as well as how formulation properties elicit meaning and beliefs from the end user (e.g., [30]). Understanding and targeting well-defined product properties will minimize expensive clinical testing on flawed designs and optimize willingness to try these novel and potentially life-saving products.

## Abbreviations

API: Active pharmaceutical ingredient
CAPRISA: Centre for the AIDS programme of Research in South Africa
FACTS: Follow-on African Consortium for Tenofovir Studies
HAI: Heterosexual Anal Intercourse
HIV: Human Immunodeficiency Virus
KCl: Potassium Chloride
STD: Sexually transmitted disease
STI: Sexually transmitted infection
US: United States
VOICE: Vaginal and Oral Interventions to Control the Epidemic
